# The effect of a theory-based educational program on southern Iranian prisoners’ HIV preventive behaviors: a quasi-experimental research

**DOI:** 10.1186/s12889-022-13763-z

**Published:** 2022-07-14

**Authors:** Zahra Hosseini, Pirdad Najafi, Shokrollah Mohseni, Teamur Aghamolaei, Sara Dadipoor

**Affiliations:** 1 Social Determinants in Health Promotion Research Center, Hormozgan Health Institute, Hormozgan University of Medical Sciences, Bandar Abbas, Iran; 2 Student Research Committee, Hormozgan University of Medical Sciences, Bandar Abbas, Iran; 3 Cardiovascular Research Center, Hormozgan University of Medical Sciences, Bandar Abbas, Iran; 4 Infectious and Tropical Diseases Research Center, Hormozgan Health Institute, Hormozgan University of Medical Sciences, Bandar Abbas, Iran

**Keywords:** AIDS, HIV, Health belief model, Prisoners

## Abstract

**Background:**

The present research explored the effect of an educational program based on the health belief model (HBM) on prisoners’ HIV preventive behaviors in the south of Iran.

**Methods:**

The present quasi-experimental research was conducted in 2019–20 on 280 prisoners, 140 in the control group (CG) and 140 in the intervention group (IG). The sampling was simple randomized. The data were collected using a questionnaire in two parts, one exploring the demographic information and the other the HBM constructs. The final follow-up was completed 3 months after the educational intervention (8 sessions long) in November 2020.

**Results:**

After the intervention, statistically significant between-group differences were found in the healthy behavior score and all HBM constructs except for the perceived barriers (*p* < 0.001). Perceived severity and susceptibility were found to be the strongest predictors of HIV preventive behaviors.

**Conclusion:**

The educational intervention showed to positively affect the adoption of preventive behaviors mediated by the HBM constructs. To remove barriers to HIV preventive behaviors or any other healthy behavior, researchers are suggested to develop multi-level interventions (beyond the personal level) to gain better findings.

**Supplementary Information:**

The online version contains supplementary material available at 10.1186/s12889-022-13763-z.

## Background

HIV, the virus accounting for the acquired immunodeficiency syndrome (AIDS), is one of the world’s most serious health issues. Approximately 38 million people are currently living with HIV. Tens of million people have died of AIDS-related reasons since the beginning of the epidemic [1]. Despite a global decline in the prevalence of the new HIV infection, in countries such as Iran, the rate of HIV infection is still high. In 2019, the estimated number of HIV patients in Iran was 59,000. Every year, about 4100 new infected cases are diagnosed, and 2500 AIDS-related mortalities occur in the country [2]. Among different populations, prisoners are at a higher risk of HIV, HCV, and HBV infections due to high-risk behaviors such as drug abuse and unprotected sex [3]. . Prisoners are 7 to 12 times more likely to be infected with HIV than the public [4]. The outbreak of the disease in Kermanshah Prison in 1995 triggered a national response to HIV [2].

Among the estimated 10.2 million prisoners world-wide, 3.8% were found to be HIV-positive [5]. A systematic review/meta-analysis of prisoners in 2019 showed an incidence rate of 0 (in Bosnia and Herzegovina) to over 20% (in Iran, Zambia, Spain) [6]. Similarly, in another systematic review, the prevalence of HIV was found to be between 0 and 24.40% among Iranian prisoners [7]. In two other studies, the same rate was reported to be 1.23 and 2.1% [8, 9].

Specific policies are made to prevent and control HIV infection in the prisons of Iran. Examples are screening the newly admitted prisoners, distributing condoms for safe sexual activities [10], initiating and expanding the administration of methadone maintenance therapy, setting up triangular clinics in prisons and exchanging needle/syringe regularly [9, 11].

Prisons are hazardous places for HIV infection due to the overcrowd, poor nutrition, limited healthcare, continued drug abuse, unsafe injections, unprotected sex and tattoos. In addition, many prisoners come from marginalized populations – such as the injecting drug users (IDU), who have already experienced an elevated risk of HIV [12].

As suggested by the World Health Organization (WHO), the best way to control HIV is to educate populations that are more at risk [13]. . There is research evidence that health education and knowledge promotion are the best ways to fight AIDS before it grows any further [14]. The HBM is a disease prevention model with a primary focus on how belief and behavior go hand in hand. It assumes that showing preventive behaviors depends on people’s perceived risk of the disease, the effect of the disease on their life and the effect of healthy behaviors on less susceptibility to and severity of the disease [15]. HBM constructs can apply to HIV educational programs, and raise awareness of HIV preventive behaviors [16]. A body of research has proved the effectiveness of HBM-based educational interventions in preventing HIV in different populations [14, 17]. This model has six constituent parts including perceived susceptibility, severity, benefits, barriers, self-efficacy and cues for action [18].

Educational interventions have been previously used in relation to HIV. Yet, they mostly addressed populations other than prisoners, or they used other theories than the HBM [12]. Maintaining prisoners’ health protects a whole society. Thus, HIV preventive measures are essential in prisons to provide useful education and information [6]. The present research is pioneering in exploring the effect of an educational program based on the HBM on prisoners’ HIV preventive behaviors in the south of Iran. The present findings suggest useful strategies to implement educational interventions and promote HIV preventive behaviors to health policy-makers.

## Methods

### Design and population

The present research was quasi-experimental in type. There were two groups included, a control (CG) and an intervention group (IG). The research was done in 2019–20 with an educational program developed based on the HBM to promote HIV preventive behaviors in prisoners with 3–5 years’ imprisonment in the south of Iran. A 3-month follow-up was also included.

### Setting

The present research was set in Roudan County in the south of Iran, with an area of about 3044.4 km^2^. Roudan is 100 km away from Bandar Abbas. Its capital city with the same name, Roudan, is located in 27°:27′ of the north and 57°:11′ of the east at an altitude of about 190 m above the sea level.

### Eligibility criteria

The inclusion criteria were: at least 6 months’ time left until release from the prison, no chronic mental disease (according to the medical records) and informed consent to participate in the research.

### Exclusion criteria

The exclusion criteria were failure to regularly attend the educational sessions (absence for more than 2 sessions), not to be available for the post-test, and incomplete questionnaires.

### Sample size estimation

To estimate the sample size, the following formula was used:

$${n}_1={n}_2=\frac{{\left({z}_{1-\frac{\alpha }{2}}+{z}_{1-\beta}\right)}^2\left({\delta_1}^2+{\delta_2}^2\right)}{{\left({\mu}_1-{\mu}_2\right)}^2}$$

In their study, Ebrahimipour et al. reported the standard deviation of self-efficacy in the intervention and control groups as 13.24 and 15.32, respectively [12]. They assumed α to be 0.05, β as 0.2 and *μ*_1_- *μ*_2_ as 5. Thus, they estimated a sample size of 130. With an attrition rate of 5–7%, the final sample size was decied to be 140.

### Sampling

**Fig. 1:**
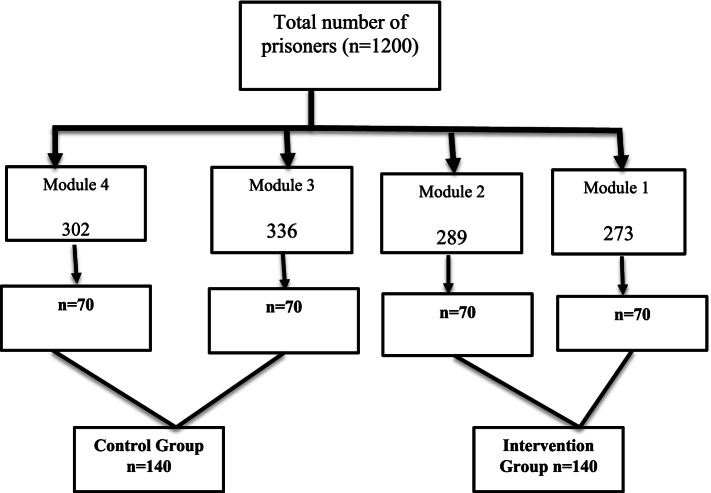
Flowchart for sample selection

There are 4 modules (or pods) in Shahid Lajevardi Prison in Roudan. There are 1200 inmates overall (i.e., about 300 in each module). The 1st and 2nd modules, which were adjacent, were selected as the IG and the 3rd and 4th as the CG. There are certain educational and cultural activities routinely planned in this prison for inmates. Modules 1 and 2 receive the educational and cultural services on different days from the modules 3 and 4. Thus, we decided to include modules 1 and 2 together in one group and modules 3 and 4 in the other group. The list of inmates in all four modules was obtained from the authorities. The Excel software was used to select 70 subjects from each module through simple randomization. If a subject did not meet the inclusion criteria, he was replaced by another through simple randomization (Fig. 1). To ensure the minimal contamination effect, the IG and CG subjects were selected from different modules. Thus, the inmates had fewer chances of communicating with each other. The break time of the two groups was scheduled to be different from each other.

### Intervention procedure

The pretest was given to the CG and IG using the HBM questionnaire. According to the pretest results, an educational need analysis was done to decide on the educational materials, methods and number of sessions needed for education. The educational content of each session was decided on according to the learners’ comprehension, use of reliable scientific sources, experts’ commentaries as well as those of the participants within the HBM framework. Besides the target behaviors, the educational methods, number of sessions and duration of each session were specified in the material development process. Overall, 8 educational sessions were to be held for 2 months in 10–15 educational groups. Each session was 40 to 60 minutes long with a 10-minute break. The teaching modes were lecture, group discussions, brainstorming, concept mapping, movies, and photos. It is noteworthy that all subjects participated until the last session, and there was no attrition. The CG had a 1-hour educational session on HIV transmission and the significance of personal health in preventing the infection. Three months after the intervention, the posttest questionnaire was given to both groups to assess the effectiveness of the educational intervention.

The educational content included:

1. general considerations about HIV and some facts and figures about the incidence rate,
2. emphasis on the hazardous prison environment and how it affected HIV infection,
3. prisoners’ awareness of the different ways of transmitting the disease and high-risk behaviors in prison,
4. physical, mental and social benefits of no HIV infection,
5. challenges of and barriers to HIV preventive behaviors, and increased self-efficacy.

The details of the educational sessions are summarized in Supplementary 1.

### Data collection

The data were collected as self-reporting questionnaires. Having consented to take part in the study, the participants in each group received adequate information about the purpose of study and what they were expected to do. The pre-test questionnaires were provided to the IG and CG. Also, 3 months after the educational intervention, the post-test questionnaires were provided to both groups. For the participants who were illiterate, the questions were read out loud by one of the researchers to minimize the bias. A specific well-trained member of the research teach was assigned to this task. The answers were transcribed with no change or personal interpretation. The questionnaire completion took between 20 and 25 minutes.

### Questionnaire content and scoring system

The questionnaire contained closed-ended questions rated on a Likert scale. There were two parts as introduced below.

### Part I (demographic information)

Several variables were included in this part of the questionnaire. These included the participants’ age, level of education, marital status, job, history of imprisonment, history of drug abuse, the use of condoms in sex affairs, and sex partners.

### Part II (HBM constructs)

The HBM constructs are summarized in Table 1. The content of the questionnaire is presented in Supplementary 2.

**Table 1:** Description of the research instrument

Constructs	No. of Items (scale)	Scoring (Range)	Internal consistency (Cronbach’s alpha)	Sample item
Perceived susceptibility	5 items (Likert Scale Questions)	Strongly Disagree = 1, Disagree = 2, No idea = 3, Agree = 4, Strongly Agree = 5	0.86	Prison is a hazardous environment and if I do not take enough care I may get infected with HIV.
Perceived severity	6 items (Likert Scale Questions)	Strongly Disagree = 1, Disagree = 2, No idea = 3, Agree = 4, Strongly Agree = 5	0.85	If I am infected with HIV, I may die sooner than expected.
Perceived Benefits	5 items (Rating Scale Question)	Strongly Disagree = 1, Disagree = 2, No idea = 3, Agree = 4, Strongly Agree = 5	0.78	Using protectives in sex affairs prevents the infection with the disease.
Perceived Barriers	7 items (Likert Scale Questions)	Strongly Disagree = 1, Disagree = 2, No idea = 3, Agree = 4, Strongly Agree = 5	0.86	It is hard to access disposable syringes in prison
Self-efficacy	5items (Likert Scale Questions)	Strongly Disagree = 1, Disagree = 2, No idea = 3, Agree = 4, Strongly Agree = 5	0.80	I can use disposable syringes for injection of drugs.
Behavior	8 Item (Numeric Text Question)	Strongly Disagree = 1, Disagree = 2, No idea = 3, Agree = 4, Strongly Agree = 5	0.84	I avoid anal sex without any protectives.

All items were rated on a 5-point Likert scale: strongly agree (1 point), agree (2 points), neutral (3 points), disagree (4 points), and strongly disagree (5 points). Each construct was assessed separately, and the total score was not calculated. The score for each construct was calculated for each participant. Higher scores represented stronger feelings about that construct. All constituent parts showed to be positively associated with the target behavior except for the perceived barriers which was negatively correlated.

### Data quality assurance

The researcher-made instrument was developed in the light of the related literature, and the national plan to prevent and control HIV infection developed by the ministry of health and the deputy of health in the disease management center. Before the main data collection phase, the questionnaires were piloted on a group of 20 subjects similar to the main participants. Their comments were used to revise the content of the questionnaire and better organize the items. The content validity was also approved by a panel of experts. Then, the required qualitative and quantitative adaptations were made. The internal consistency of the instrument was approved using Cronbach’s alpha. To substantiate the reliability of the questionnaire, the test-retest method was used. To this aim, the questionnaire was submitted twice at a 2-week interval to 20 subjects who were similar to the main participants. The ICC was found to be 0.86, interpreted as high. Thus, the reliability of the questionnaire was confirmed.

### Ethical considerations

The participants were supposed to sign an informed letter of consent. The confidentiality of the information they provided was ensured. All the required measures were taken to ensure the confidentiality of the participants’ information. The research procedures were fully explained. The results were also, later on, provided to them. Prisoners with mental diseases were more vulnerable. Their condition could affect their voluntary decisions. Thus, their decision whether to participate in the study or not was respected. Their decision did not affect the availability of facilities provided in prison, such as healthcare services or healthy food. If they did not consent to take part in the research, they were not treated adversely by the prisoners. Their participation was quite fair and respected. Before the study, the final draft of the questionnaire was reviewed by the prison authorities, and their comments were used to revise the instrument.

The study conformed to the world medical association (WMA) of Helinski and the Nuremberg Code. The project was approved by the ethics committee of Hormozgan University of medical sciences (#IR.HUMS.REC.1398.112).

#### Output

The output was an increase in perceived susceptibility, severity, benefits, self-efficacy and barriers.

#### Outcome

The expected outcome was the adoption of HIV preventive behaviors.

### Data management and analysis

To analyze the quantitative variables (age and HBM scores), mean and standard deviation were used. To describe qualitative data, frequency and relative frequency were used. To test the assumptions of parametric tests such as the normality of distribution and equality of variance, Kolmogorov-Smirnov test and Levene’s test were run. Then, independent-samples *T*-test was used to compare HBM scores and the adoption of preventive behaviors in the two groups. Paired-samples *T*-test was run to compare the pretest and post-test results within each group. ANCOVA was used to control and adjust for the scores before and after the intervention. Besides, multiple linear regression analysis was run to assess the effect of each HBM construct on the behavior score. Healthy behavior was considered as the dependent variable and the model constructs as independent variables. All the analyses were done in SPSS20.

## Results

### Research population

The present quasi-experimental study was conducted on a total number of 280 prisoners (140 in the IG and 140 in the CG). The participants’ age ranged between 19 and 65 years with a mean and standard deviation of 35.49 ± 8.24 in the CG and 35.29 ± 8.82 in the IG. Concerning education, in both research groups, the most frequent education level was secondary school (39.3% in the IG and 40.03% in the CG). The majority of prisoners in both groups had 1–2-time experience of imprisonment (94.35% in the IG and 80% in the CG). The majority of prisoners had a history of drug addiction (57.9% in the IG and 67.1% in the CG). The other demographic variables are summarized in Table 2.

**Table 2:** Research participants’ demographic information

Variable	category	Total sample N (280)	Intervention group (***n*** = 140)	Control group (***n*** = 140)	***p***-value
Age (M,SD)		35.39 (8.25%)	35.49 (8.24%)	35.29 (8.823%)	0.850
Educational level	Illiterate	11 (3.9%)	3 (2.1%)	8 (5.73%)	0.401
	primary	76 (27.1%)	43 (30.7%)	33 (23.63%)	
	Secondary	111 (39.6%)	55 (39.3%)	56 (40.03%)	
	Diploma	71 (25.4%)	33 (23.63%)	38 (27.13%)	
	College	11 (3.9%)	6 (4.33%)	6 (3.63%)	
Marital status	Single	86 (30.7%)	45 (32.13%)	41 (29.33%)	0.256
	Married	167 (59.6%)	78 (55.73%)	89 (63.63%)	
	Divorced/widowed	27 (9.6%)	17 (12.13%)	10 (7.13%)	
occupation	unemployed	56 (20.0%)	29 (20.73%)	27 (19.33%)	0.694
	Manual jobs	133 (47.5%)	70 (50.03%)	63 (45.03%)	
	farming	66 (23.6%)	29 (20.73%)	37 (26.43%)	
	other	25 (8.9%)	12 (8.63%)	13 (9.33%)	
History of imprisonment	1–2	244 (87.1%)	132 (94.33%)	112 (80%)	0.000
	3 or more	36 (12.9%)	8 (5.73%)	28 (20%)	
History of drug addiction	yes	175 (62.5%)	81 (57.93%)	94 (67.1%)	0.109
	No	105 (37.5%)	59 (42.13%)	46 (32.9%)	
Using protectives in sex affairs with one’s spouse	yes	46 (16.4%)	21 (15.03%)	25 (17.9%)	0.766
	no	148 (52.9%)	74 (52.93%)	74 (52.9%)	
	Not married	86 (30.7%)	45 (32.13%)	41 (29.3%)	
Physical contact with a partner (other than the spouse)	yes	134 (47.9%)	65 (46.43%)	69 (49.3%)	0.632
	no	146 (52.1%)	75 (53.63%)	71 (50.7%)	
Using protectives in sex affairs with one’s sex partner	yes	65 (23.2%)	29 (20.73%)	36 (25.7%)	0.608
	no	69 (24.6%)	36 (25.73%)	33 (23.6%)	
	No sex affair	146 (52.1%)	75 (53.6%)	71 (50.7%)	

### Between-group comparison of HBM constructs in the pretest and posttest

Before the intervention, the two groups showed no statistically significant difference in terms of the HBM scores (*p* > 0.05). However, after the educational intervention, the between-group difference was statistically significant (*p* < 0.001). In the IG, the behavior score was 22.93 ± 4.35 in the pretest, which was increased to 31.85 ± 0.739 in the posttest. This increase was statistically significant. However, in the CG, the behavior score did not change significantly from the pretest to posttest (Table 3).

**Table 3:** Between-group comparison of HBM constructs in the pretest and posttest

Variables	Group	Pretest (before intervention) (Mean ± SD)	posttest (after intervention) (Mean ± SD)	***P***-value
Perceived susceptibility	Intervention	18.59 ± 4.20	24.39 ± 1.63	**0.001>**
	Control	18.41 ± 3.39	19.09 ± 3.88	**>** 0.112
	*P*-value	**0.698**	**0.001>**	
Perceived severity	Intervention	20.77 ± 5.13	29.19 ± 1.97	**0.001>**
	Control	21.84 ± 5.07	22.47 ± 4.93	0.164**>**
	*P*-value	**0.082**	**0.001>**	
Perceived barriers	Intervention	22.41 ± 5.47	19.76 ± 3.20	**0.001>**
	Control	21.72 ± 5.68	20.58 ± 6.39	**0.070>**
	*P*-value	**0.299**	**0.179**	
Perceived benefits	Intervention	21.69 ± 6.16	29.24 ± 1.99	**0.001>**
	Control	22.11 ± 4.94	23.04 ± 4.63	0.109**>**
	*P*-value	**0.522**	**0.001>**	
Self-efficacy	Intervention	17.40 ± 4.22	24.04 ± 1.94	**0.001>**
	Control	17.05 ± 4.73	17.84 ± 4.50	**0.127>**
	*P*-value	**0.514**	**0.001>**	
Behavior	Intervention	22.93 ± 4.35	31.85 ± .739	**0.001>**
	Control	23.26 ± 5.77	24.03 ± .6.369	**0.059>**
	*P*-value	**0.591**	**0.001>**	

### Controlling the covariate effect of scores in the pretest

To control and adjust for the effect of pretest scores, ANCOVA was used. As summarized in Table 3, the pretest scores were found to be statistically significant covariates of perceived severity (partial η2 = 0.084; *p* = 0.001), perceived barriers (partial η2 = 0.036; *p* = 0.002), and behavior (partial η2 = 0.370; *p* < 0.001). But as the pretest scores and the ANCOVA result showed, perceived susceptibility (partial η2 = 0.001; *p* = 0.692), benefits (partial η2 = 0.001; *p* = 0.825) and self-efficacy (partial η2 = 0.001; *p* = 0.534) were not statistically significant.

#### Predictors of AIDS preventive behavior

To analyze the effect of each HBM construct on the adoption of healthy behavior, multiple linear regression analysis was used. How the dependent and independent variables behaved was different. As indicated in Table 4, perceived severity, susceptibility, benefits, self-efficacy and barriers were the best predictors of healthy preventive behavior. The adjusted R-square of 0.411 shows that the model managed to explain 41% of variation in behavior in the intervention group (Table 5).

**Table 4:** Analysis of covariance to adjust the pre-intervention scores as the covariate

Variables	Source	Sum of Squares	df	Mean Square	statistic F	***p***-value	Partial Eta Squared
**Perceived susceptibility**	baseline score	1.40	1	1.40	.157	.692	.001
	intervention	1968.050	1	1968.050	220.623	.000	.443
	error	2470.960	277	8.920			
	R Squared = .444 (Adjusted R Squared = .440)						
**Perceived severity**	baseline score	331.328	1	331.328	25.513	.000	.084
	intervention	3343.666	1	3343.666	257.466	.000	.482
	error	3597.351	277	12.987			
	R Squared = .493 (Adjusted R Squared = .489)						
**Perceived benefits**	baseline score	.627	1	.627	.049	.825	.000
	intervention	2689.980	1	2689.980	210.166	.000	.431
	error	3545.416	277	12.799			
	R Squared = .432 (Adjusted R Squared = .427)						
**Perceived barriers**	baseline score	252.736	1	252.736	10.201	.002	.036
	intervention	60.665	1	60.665	2.449	.119	.009
	error	6862.621	277	24.775			
	R Squared = .042 (Adjusted R Squared = .035)						
**Self-efficacy**	baseline score	4.676	1	4.676	.388	.534	.001
	intervention	2677.917	1	2677.917	221.984	.000	.445
	error	3341.610	277	12.064			
	R Squared = .446 (Adjusted R Squared = .442)						
**Behavior**	baseline score	2116.404	1	2116.404	162.966	.000	.370
	intervention	4473.805	1	4473.805	344.490	.000	.554
	error	3597.332	277	12.987			
	R Squared = .640 (Adjusted R Squared = .638)

**Table 5:** Predictors of AIDS preventive behavior based on the HBM model

Variables	B	95.0% Confidence Interval for B		Standardized Coefficients Beta	t	*p*-value
		Lower Bound	Upper Bound			
Perceived susceptibility	.289	.112	.466	.193	3.211	.001
Perceived severity	.536	.384	.687	.451	6.97	0.001>
Perceived benefits	.232	.091	.373	.183	3.24	0.001>
Perceived barriers	−.117	−.228	−.006	−.099	−2.082	.038
Perceived Self-efficacy	.161	.005	−.006	.125	2.035	.005

R Square = 0.400 Adjusted R Square = 0.411

## Discussion

The present study explored the effect of an educational intervention on the adoption of HIV preventive behaviors based on the HBM model. Multivariate regression analysis (R2 = 0.411) showed that the independent variable in the model (HBM constructs) managed to explain 41% of variance in the dependent variable (i.e. adoption of HIV preventive behavior).

The present findings showed that the two groups did not diverge significantly in terms of perceived susceptibility before the intervention. However, after the educational intervention, the between-group difference was statistically significant. This finding was consistent with a body of research that showed the effectiveness of educational interventions in increasing the chances of HIV infection [14, 17, 19]. However, a number of studies reported the failure of educational interventions at increasing participants’ susceptibility of HIV and medical adherence in HIV patients [20–22]. This divergence can be partly due to the differing demographic features of the research populations. In the abovementioned studies, the research population was female adolescents, often at a lower risk of high-risk behaviors such as sexual behaviors and drug injection than the target population in this research. Further divergences can be the duration, content and teaching methods used in the intervention, which were more limited in the aforementioned studies than the present study. It is noteworthy that perceived susceptibility showed to affect prisoners’ promotion of healthy behavior. Arguably, the theory-based education managed to increase prisoners’ susceptibility to the infection. Researchers believe that, to motivate a certain healthy behavior, people need to get aware of the potential adverse effects of a disease or how it affects their awareness [23].

The present findings showed that the mean score of perceived severity was increased in the IG. Similarly, a body of research showed that educational interventions managed to increase the mean score of perceived severity in [14, 16, 19, 24]. Moreover, our findings showed that perceived severity was the strongest predictor of adopting HIV-AIDS preventive behaviors. This is in contrast to some other research which showed no effect of perceived severity on the adoption of healthy behavior [24]. It can be argued that the severely adverse effects of HIV infection are adequately perceived by the prisoners. Thus, prisoners are motivated enough to show HIV preventive behaviors. Presumably, prisoners with a better perceived severity of the adverse effects of HIV show more protective behaviors. According to Rosen Stock’s theory, perceived severity can promote preventive and medical measures in individuals [25]. As put forth by Bakhtiari, one who perceives him/herself at the risk of a major problem, takes a serious measure to protect oneself [26].

The present findings showed that the mean score of perceived benefits was significantly increased in the IG compared to the CG. Similarly, a body of research reported the effectiveness of education in increasing the perceived benefits of HIV preventive behaviors [14, 16, 27] . Contrary to the present findings, in a number of studies, perceived benefits was not correlated with HIV preventive behaviors [22, 28] . Different purposes of research and socio-demographic features in different geographies can be other potential reasons for the different findings. As an instance, in the study conducted by Gharlipour et al., probably failed HIV therapeutic measures canceled out the effect of the educational intervention on the participants’ perceived benefits. Our educational intervention, however, evidently highlighted the benefits of preventing HIV and managed to encourage people to adopt preventive healthy behaviors.

We also found that the educational intervention had no effect on perceived barriers. This is consistent with a number of studies that reported the ineffectiveness of educational interventions in HIV preventive behaviors and adherence to medications [14, 22]. Contrary to this finding, some other studies found an increase in perceived barriers after the educational intervention [20, 27, 29]. Different types of barriers in different studies (physical, financial, psychological and social) can also account for the divergent findings. No increase in the perceived barriers score in the present study was quite expected because, as also reflected in the questionnaire items, most barriers were out of an individual’s control. Naturally, in only one educational intervention, we were unable to overcome such personal barriers that required higher-order interventions such as organizational, social and even political. Of note is that in this research, a lower perceived barrier score was accompanied by a higher rate of healthy behaviors. Thus, it can be expected to be effective in the adoption of healthy behavior.

The present findings also revealed a higher mean score of self-efficacy in the IG than CG in the posttest. This is consistent with a number of studies which also reported an increase in the self-efficacy score after the educational intervention [14, 27]. Another study showed that self-efficacy was significantly and strongly correlated with HIV preventive behaviors in Thai youngsters [12]. Furthermore, self-efficacy has proved to be key to the reduced rate of high-risk AIDS-related behaviors [30]. In contrast, in two other works of research by Smith and Bandora [31]. and Zamboni [32]., education showed to have no effect on patients’ self-efficacy [22]. Improving self-efficacy was suggested as a secondary goal for lowering the rate of HIV infection. According to the socio-cognitive theory, those with a lower self-efficacy stand higher chances of showing risky behaviors [27]. As expected in our research, those with a higher self-efficacy showed more HIV-AIDS preventive behaviors [28]. Thus, improving prisoners’ self-efficacy can to a large extent prevent the incidence rate of HIV.

The present findings showed an increase in the participants’ score of HIV preventive behaviors in IG compared to CG after the intervention. Similarly, other studies reported the effectiveness of adopting HIV preventive behaviors and adherence to medications [16, 22] . It can be argued that the educational intervention could have positively affected the participants’ healthy behavior by affecting the HBM constructs as the mediating factors.

### Limitations, strengths and suggestions for future research

The present research was conducted on male prisoners in the south of Iran; thus, the generalization of the findings to other populations especially women is limited. To increase the generalizability, future research needs to include comparable male and female samples in areas with different cultural and socioeconomic features. The short-term follow-up was another limitation. Therefore, it is suggested that the participants be followed up for at least a year to assess their consistency of behavior. Another limitation of this research was the self-reporting nature of the questionnaire. The participants might have produced socially desirable responses which can threaten the integrity of responses. Still, we attempted to ensure the subjects of the confidentiality of the information they provided to maximize their honesty. The data were collected anonymously to reduce the biased responses. A lack of access to confidential prison information, including the number of HIV-infected inmates and drug abuse in prisons, were among the other limitations of the present study.

There were certain strengths as well. For instance, the theory employed (i.e., the HBM) was a systematic framework to explain the healthy preventive behavior. This theory clearly described the key concepts included in the intervention [33]. Making a goal-oriented and theory-based intervention, selecting a high-risk research population and having a control group are among the other strengths of the present research.

### Implications

As there is no definite cure for HIV infection and there has been no theory-based educational intervention for the target research population (i.e., prisoners), the present findings can significantly contribute to the existing literature. They pave the way for future comparative HIV-related research and can help policy makers develop better interventional programs to prevent HIV-related risky behaviors in the light of relevant theories.

## Conclusion

The present research showed the effectiveness of HBM in adopting HIV preventive behaviors among prisoners. The educational intervention managed to positively affect the prisoners’ healthy behaviors by affecting the HBM constructs first. As the results showed, the educational intervention had no effect on perceived barriers, which was quite expected, as perceived barriers could not be removed until the end of a simple short-term intervention. To remove barriers to the adoption of healthy behaviors, researchers should develop multi-level interventions to gain more desirable outcomes. We particularly aim to implement goal-oriented educational programs based on health education and promotion frameworks to prevent HIV behaviors.

## Supplementary Information

**Additional file 1.** Education and training content.

**Additional file 2.**

## Data Availability

The datasets used and/or analyzed during the study are available from the corresponding author on reasonable request.

## Abbreviations

HBM: Health belief model
IG: Intervention group
CG: Control group
