# Supplementary material for: The effect of a theory-based educational program on southern Iranian prisoners’ HIV preventive behaviors: a quasi-experimental research
Source: BMC Public Health. 2022 Jul 14;22:1342. doi: 10.1186/s12889-022-13763-z (PMC9281156; doi:10.1186/s12889-022-13763-z)
Supplement: Supplementary file 1 — Additional file 1. Education and training content. [file 12889_2022_13763_MOESM1_ESM.docx]

**Supplementary file 1. Education and training content**

| **Session/class** | **content** | **activities** |
| --- | --- | --- |
| Week 1  60 minutes | Familiarity with the purpose of study and the educational intervention, expectations and the process of implementing the educational intervention, gaining the participants’ trust | In this session, the teacher acquainted the participants with the purpose and significance of study. Participants learned about teaching methods and syllabus based on a pre-developed checklist. The content was then revised according to the participants’ feedback. |
| Week 2  60 minutes | Familiarity with HIV infection, all details and symptoms of HIV | In this session, the prisoners received the instructions through lectures, brainstorming, active participatory discussion and using images through OHPs and videos about HIV, as well as statistics on the prevalence and incidence of HIV at a national and global scale especially in the Islamic Republic of Iran. The disease was also described in detail.  At the end of the class, an evaluation checklist was completed to identify deficiencies and remove them. |
| Week 3  40 minutes | Familiarity with the ways of transmitting HIV | In this session, the prisoners were familiarized with the effect of high-risk behaviors in increasing HIV infection and transmission through lectures, active participatory discussions and brainstorming. |
| Week 4  60 minutes | Familiarity with the risk factors of HIV infection | In this session, the prisoners were introduced to the risk factors for HIV infection through lectures, active participatory discussions and brainstorming. At the end of the session, an evaluation checklist was completed to identify deficiencies and solve them. |
| Week 5  60 minutes | Adverse effects of HIV | In this session, the prisoners were introduced to the following content through lectures, active participatory discussions and brainstorming.  1. High risk of prison environment and its effect on increasing HIV infection  2. Possible consequences of HIV infection, such as social stigmatization (caused by the disease), discrimination and exclusion in family and friends; the effect of HIV on life expectancy, disease-related death and the psychological impact of the disease on one’s life |
| Week 6  60 minutes | Familiarity with risky behaviors, benefits of avoiding risky behaviors, barriers to the adoption of healthy behaviors | In this session, the prisoners were introduced to the following items by the teacher through lectures, active participatory discussions, brainstorming and role play:   - High-risk behaviors in prison and the benefits of avoiding high-risk behaviors - The effect of preventive behaviors (by identifying and removing the barriers) in showing healthy behavior   Prisoner trainees were asked to re-write their suggestions for removing barriers to prison-related behaviors. |
| Week 7  40 minutes | Remove barrier through increasing self-efficacy | In this session, the content of previous sessions was reviewed.  Barriers to healthy behavior were discussed to reduce the transmission of HIV as perceived by prisoners with the aim of increasing self-confidence.  The benefits of self-efficacy were discussed along with the fact that they are capable of preventing HIV through a well-organized plan. |
| Week 8  60 minutes | Continue to remove barriers by increasing self-efficacy | The pretest and also prisoners’ facial expressions in previous instructional sessions showed there were different barriers to preventive behaviors. There was a strong need for removing the barriers. Therefore, the following was done to remove the barriers.  The training was conducted in the presence of prison officials, the judiciary, the city health center and influential people in removing barriers and tackling the issues with preventive behaviors in prison.  During this session, the prisoners reported their problems to the people in charge and asked for their solution.  Later, it was found that the training session and the invitation of the officials had a significant effect on increasing preventive behaviors. |
