# Supplementary material for: The effect of a theory-based educational program on southern Iranian prisoners’ HIV preventive behaviors: a quasi-experimental research
Source: BMC Public Health. 2022 Jul 14;22:1342. doi: 10.1186/s12889-022-13763-z (PMC9281156; doi:10.1186/s12889-022-13763-z)
Supplement: Supplementary file 2 — Additional file 2. [file 12889_2022_13763_MOESM2_ESM.docx]

**Dear respondent**

The present questionnaire survey is an academic study on your health behaviors. The results will be used for the educational planning of prisoners. There is no need to mention your name, and your answer to the questions will be kept completely confidential. Thank you in advance for your honest participation and response.

**Part I: Demographic information and behavioral background**

1. Age (In years) …………..
2. Education level: a. illiterate □ b. elementary school □ c. junior high school □ d. high school (diploma) □ e. university □
3. Marital status: a. single □ b. married □ c. divorced □ d. widowed □ e. other □ (please explain ………………….)
4. Job: a. unemployed □ b. blue-collar worker □ c. farmer □ d. white-collar worker (public sector) □ e. white-collar worker (private sector) □ f. other □ (please explain …………….)
5. How often have you already been imprisoned? ………… times
6. Any history of drug abuse? Yes □ no □
7. Do you wear a condom in sex affairs with your spouse? A. yes □ b. no □
8. Have you have any sex affair with someone other than your spouse? A. yes □ b. no □
9. Have you worn a condom in the sex affair with a partner other than your spouse? A. yes □ b. no □

**Perceived susceptibility**

| Please mark to what extent you agree with each of the following statements. | Strongly agree | Agree | No idea | Disagree | Strongly disagree |
| --- | --- | --- | --- | --- | --- |
| 1. Prison is a dangerous environment, and if I am not careful, I could get AIDS. |  |  |  |  |  |
| 1. Once I have sex without a condom, I may get AIDS. |  |  |  |  |  |
| 1. Anal intercourse is more likely to transmit AIDS. |  |  |  |  |  |
| 1. Injections (drugs) with a shared syringe and tattooing with a shared needle increase my risk of contracting AIDS. |  |  |  |  |  |
| 1. Damage to my skin with sharp objects such as razor blades and razors, etc., increases my chances of affliction with AIDS. |  |  |  |  |  |

| Please mark to what extent you agree with each of the following statements. | Strongly agree | Agree | No idea | Disagree | Strongly disagree |
| --- | --- | --- | --- | --- | --- |
| 1. If I get AIDS, my family will reject me. |  |  |  |  |  |
| 1. If I get AIDS, my friends in prison will abandon me. |  |  |  |  |  |
| 1. If I get AIDS, I may die sooner. |  |  |  |  |  |
| 1. If I get AIDS, I will have more trouble getting married and taking care of my spouse and children. |  |  |  |  |  |
| 1. If I get AIDS, my mental health problems will increase. |  |  |  |  |  |
| 1. If I get AIDS, I will not want to go back to society. |  |  |  |  |  |

**Perceived severity**

**Perceived Benefits**

| Please mark to what extent you agree with each of the following statements. | Strongly agree | Agree | No idea | Disagree | Strongly disagree |
| --- | --- | --- | --- | --- | --- |
| 1. Wearing a condom during sexual intercourse prevents me from getting AIDS. |  |  |  |  |  |
| 1. Using a disposable razor and razor will prevent me from getting AIDS. |  |  |  |  |  |
| 1. By showing healthy behaviors and not getting infected with AIDS, I can still maintain my family and friends. |  |  |  |  |  |
| 1. I will have more peace of mind by showing AIDS preventive behaviors. |  |  |  |  |  |
| 1. If I do not get infected with AIDS, I will be more motivated to get out of prison and return to society. |  |  |  |  |  |

**Perceived barriers**

| Please mark to what extent you agree with each of the following statements. | Strongly agree | Agree | No idea | Disagree | Strongly disagree |
| --- | --- | --- | --- | --- | --- |
| 1. It is difficult to access a condom in prison. |  |  |  |  |  |
| 1. When I wear a condom, I feel less pleasure with my partner. |  |  |  |  |  |
| 1. Disposable syringes are difficult to access in prison. |  |  |  |  |  |
| 1. Disposable razors and razors are difficult to access in prison. |  |  |  |  |  |
| 1. The price of condoms, syringes and razors is high. |  |  |  |  |  |
| 1. I do not have access to preventive treatment (prophylaxis) and prevention of AIDS if I engage in risky behavior or encounter contaminated tools in prison. |  |  |  |  |  |
| 1. The stigma of high-risk sexual behavior prevents me from expressing that behavior to prison health professionals for preventive treatment. |  |  |  |  |  |

**Self-efficacy**

| Please mark to what extent you agree with each of the following statements. | Strongly agree | Agree | No idea | Disagree | Strongly disagree |
| --- | --- | --- | --- | --- | --- |
| 1. I can avoid having a relationship with anyone other than my spouse. |  |  |  |  |  |
| 1. I'm sure I can use a condom properly if I have sex with someone other than my spouse. |  |  |  |  |  |
| 1. I can use a disposable syringe to inject the material. |  |  |  |  |  |
| 1. I can use oral methadone instead of injecting drugs. |  |  |  |  |  |
| 1. I can use disposable razors for my own cleaning purposes. |  |  |  |  |  |

**Behavior**

| Please mark how often you show each of the following behaviors. | Always | Most often | Rarely | Never |
| --- | --- | --- | --- | --- |
| 1. I avoid non-sterile and unhygienic tattoos. |  |  |  |  |
| 1. I only have sex with someone I know is healthy. |  |  |  |  |
| 1. I abstain from anal sex without a condom. |  |  |  |  |
| 1. Instead of injecting drugs, I use oral and smoking drugs. |  |  |  |  |
| 1. I use disposable syringes to inject drugs. |  |  |  |  |
| 1. I use disposable razors and razors for shaving and clean |  |  |  |  |
| 1. I go to the doctor or health consultant after having sex without a condom. |  |  |  |  |
| 1. If I have high-risk behaviors or exposure to contaminated equipment, I should contact a health professional immediately to prevent the disease. |  |  |  |  |
